# Supplementary material for: From sequence to enzyme mechanism using multi-label machine learning
Source: BMC Bioinformatics. 2014 May 19;15:150. doi: 10.1186/1471-2105-15-150 (PMC4229970; doi:10.1186/1471-2105-15-150)
Supplement: Additional file 2 — Java code of ml2db. Additional file ml2db_code.tar.gz contains the Java source code to run the multi-label machine learning experiments and save the results to database. The code’s Javadoc is included. [file 1471-2105-15-150-S2.zip › additional file 2/ml2db/utils/doc/uk/ac/standrews/utils/main/guiutils/OptionUtils.html]

OptionUtils


JavaScript is disabled on your browser.


- Overview
- Package
- Class
- Use
- Tree
- Deprecated
- Index
- Help

- Prev Class
- Next Class

- Frames
- No Frames

- All Classes

- Summary:
- Nested |
- Field |
- Constr |
- Method

- Detail:
- Field |
- Constr |
- Method


uk.ac.standrews.utils.main.guiutils

## Class OptionUtils

- java.lang.Object
- - uk.ac.standrews.utils.main.guiutils.OptionUtils

- ---

    

  ```
  public class OptionUtils
  extends java.lang.Object
  ```

  Utilities to manipulate command line options, main args etc.

  Version:
  :   4 Jan 2008

  Author:
  :   Luna De Ferrari luna.deferrari-at-ed.ac.uk

- - ### Constructor Summary

    Constructors

    Constructor and Description

    `OptionUtils()`
  - ### Method Summary

    Methods

    | Modifier and Type | Method and Description |
    | --- | --- |
    | `static boolean` | `getFlag(java.lang.String opt, java.lang.String[] options)` |
    | `static java.lang.String` | `getOption(java.lang.String opt, java.lang.String[] options)` GetOption(String opt, String[] options) gets an option value out of a list of options |

    - ### Methods inherited from class java.lang.Object

      `equals, getClass, hashCode, notify, notifyAll, toString, wait, wait, wait`

- - ### Constructor Detail


    - #### OptionUtils

      ```
      public OptionUtils()
      ```
  - ### Method Detail


    - #### getFlag

      ```
      public static boolean getFlag(java.lang.String opt,
                    java.lang.String[] options)
      ```


    - #### getOption

      ```
      public static java.lang.String getOption(java.lang.String opt,
                               java.lang.String[] options)
      ```

      GetOption(String opt, String[] options) gets an option value out of a
      list of options

      Parameters:
      :   `opt` - the option that needs to be extracted (e.g. -x)
      :   `options` - list of options (e.g. -x 10 -y name)

      Returns:
      :   the value for the option selected (e.g. 10)


- Overview
- Package
- Class
- Use
- Tree
- Deprecated
- Index
- Help

- Prev Class
- Next Class

- Frames
- No Frames

- All Classes

- Summary:
- Nested |
- Field |
- Constr |
- Method

- Detail:
- Field |
- Constr |
- Method
